# Management of multiple myeloma presenting as malignant spinal cord compression during pregnancy: a case report

**DOI:** 10.46989/001c.124664

**Published:** 2024-10-23

**Authors:** Khushnuma Mullanfiroze, Charlotte Jones, David Williams, Matias Vieira, Ashutosh Wechalekar, Xenofon Papanikolaou, Rakesh Popat, Charalampia Kyriakou, Ke Xu

**Affiliations:** 1 Haematology University College London; 2 Elizabeth Garrett Anderson Wing, Institute for Women’s Health University College London; 3 Fetal Medicine Unit University College London; 4 Elizabeth Garrett Anderson Wing, Institute for Women’s Health University College London Hospitals

**Keywords:** Multiple myleoma, Pregnancy

## Abstract

Abstract not included as this is letter to the editor.

Management of newly diagnosed multiple myeloma (MM) in pregnancy is challenging. Due to the rarity of these cases a guideline is lacking. We report a case of a 37-year-old pregnant woman who was diagnosed with MM and a malignant spinal cord compression (MSCC) in her third trimester and was successfully managed by a multi-disciplinary team (MDT).

This patient presented with left sided thoracic pain which started during the first trimester of her third pregnancy. Pleuritic chest pain progressively worsened and she developed numbness of her right toes. A computed tomography pulmonary angiography (CTPA) in the third trimester ruled out a pulmonary embolus but identified collapse of thoracic (T) 8 vertebra. At 28 weeks, magnetic resonance imaging (MRI) of the whole spine confirmed collapse of the T8 vertebrae, with an epidural soft tissue component and signs of radiological cord compression (Figure 1). T8 soft tissue biopsy confirmed plasmacytoma. She was started on dexamethasone and transferred to our haematology ward at 29+6 weeks of gestation.

**Figure 1.**
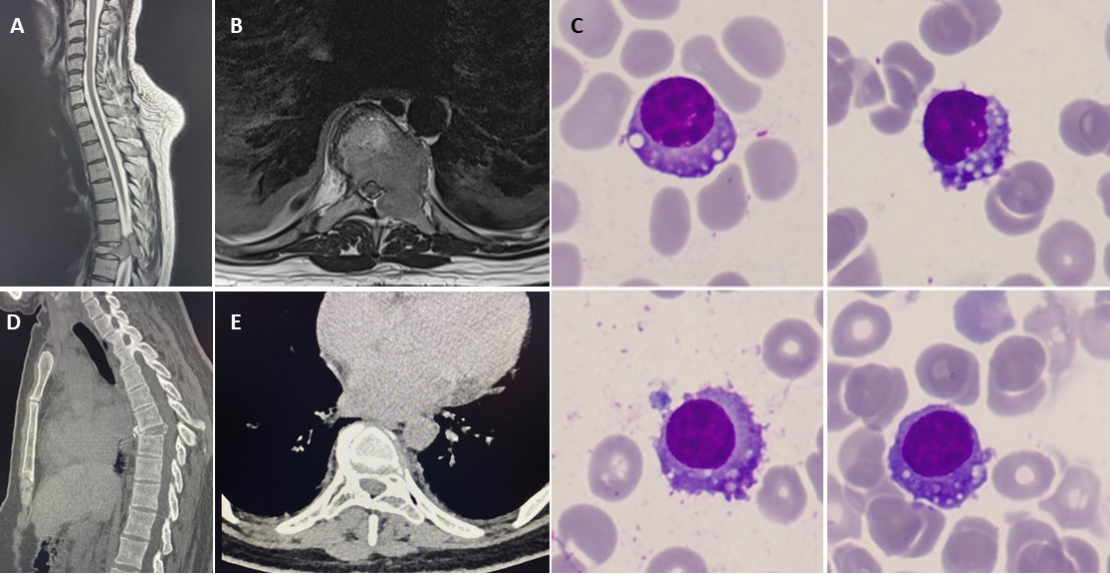
(A, B) Spine MRI showed large focal lesion involving the T8 vertebral body, left pedicle and left side of the posterior elements with pathological fracture (>50% loss of vertebral height) and extensive para-medullary disease with moderate to severe canal narrowing and cord compression. (C) Bone marrow aspirate (May-Grünwald-Giemsa stain x100 objective) showed plasma cells with frequent cytoplasmic vacuolation. (D, E) Computed tomography- single-photon emission computed tomography (CT-SPECT) post chemotherapy and radiotherapy as pre-ASCT assessment: T8 vertebral body demonstrating treated disease with new bone formation and no active disease.

At presentation, at our centre, she had no neurological signs of MSCC. She underwent an urgent staging investigation. Whole body MRI showed large T8 lesion involving the body and left posterior elements with paramedullary involvement and cord compression. There was severe loss of vertebral height and a 14mm L5 lesion. Her MM workup showed Hb 108g/L, corrected calcium 2.50 mmol/L, creatinine 45 μmol/L, LDH 168 IU/L, albumin 39 g/L, β2-microglobulin 1.5 mg/L, lambda serum free light chain of 5612 mg/L, K/L ratio <0.01; IgG paraprotein was too small to quantify. Bone marrow aspiration and trephine (BMAT) showed 30-40% clonal plasma cells (Figure 1). Fluorescent *in situ* hybridisation (FISH) on CD138-selected cells showed loss of *MAF* only. She was diagnosed with MM, standard cytogenetics risk, ISS stage 1.

An urgent MDT meeting was convened at 30 weeks of gestation. MDT attendees included haematologists, obstetric physicians, clinical oncologists, neurosurgeons, spinal orthopaedic surgeons, and anaesthetists. The following management options were discussed:

1. **MSCC management:** Though the patient had minimal neurological symptoms, with no bony component impinging the spinal cord, her spine was considered ‘unstable’. The MDT decided against neurosurgery and recommended strict bed rest with spinal precautions including log-rolling to prevent pressure ulcers, venous thrombo-embolism (VTE), gastric ulcer prophylaxis, bladder and bowel care, and adequate analgesia.

2. **Radiotherapy with uterine shielding:** Radiotherapy using modern radiation techniques and fetal shielding is now considered safe in the first two trimesters of pregnancy, especially if targeted at lesions remote from the fetus. However, in this case, the distance between the T8 lesion and third trimester fetus was considered too close to avoid significant fetal radiation exposure, and the procedure was hence disregarded.^1^

3. **Use of novel agents:** Bortezomib, a small molecule proteasome inhibitor and daratumumab, a humanised IgG1 monoclonal antibody targeting CD38, are effective MM therapies. However, it is widely accepted that the use of small molecules is contraindicated in pregnancy.^2^ An IgG1 antibody would cross the placental barrier and potentially affect the fetus.^3^ Therefore, these treatments were rejected.

4. **High-dose steroids:** Given the safety record of steroids in pregnancy and their efficacy in MM,^4,5^ the MDT recommended twice weekly pulse IV dose of 250 mg methylprednisolone. The plan was to closely monitor the patient with an aim to continue pregnancy, at least, until 34 weeks. She underwent daily detailed neurological examination, daily non-invasive fetal monitoring and weekly MRI scan of the spine. The MDT recommended that if the spinal lesion progressed or if the patient developed further neurological signs, an emergency delivery would be in her best interest. The patient was in agreement with this plan and the pregnancy was continued.

Two days later, the patient developed increasing lower limb numbness and bilateral brisk knee deep-tendon reflexes. Spine MRI done on the day showed stable disease. The methylprednisolone dose was increased to 500 mg for three consecutive days, which had good clinical benefit. The MDT recommended an elective caesarean section (C-Section) at 32 weeks of gestation, followed by palliative radiotherapy to the T8 spinal lesion, followed by cycles of systemic chemotherapy after radiotherapy, followed by high-dose melphalan autograft stem cell transplantation (ASCT).^6^

At 32 weeks, a 1,525-g (3^rd^ centile) baby girl was delivered by caesarean section under General anaesthesia (GA) with careful maternal positioning during surgery. GA was preferred over spinal anaesthesia due to concerns regarding spinal instability. The baby required respiratory resuscitation at birth, with continuous positive airway pressure (CPAP) support, and was then treated in the neonatal intensive care unit (NICU). The patient was advised about the safety of breast-feeding during radiotherapy, but that it should be avoided during chemotherapy. She therefore agreed to freeze expressed breast milk which was then fed to her baby when she started chemotherapy.

At 48-h postpartum, the patient underwent 20 Gy, 5 fraction radiotherapy to the T7-T9, which she tolerated well. Post-radiotherapy, she was allowed to mobilise wearing a thermoplastic thoraco-lumbar sacral orthosis (TLSO) brace, when upright, standing and walking.^7^ She was advised to avoid lifting, bending and twisting, and to sit at 45 degrees or lie down when the brace was removed. A week post-radiotherapy, she started systemic treatment with daratumumab, bortezomib, and dexamethasone. Thalidomide was added in cycle two with VTE prophylaxis when her mobility improved and the risk of VTE reduced. The patient made a full neurological recovery and has no residual neurological deficits or signs of spinal cord compression. Her baby was discharged from the NICU after four weeks in good health with adequate weight gain. After completion of four cycles of induction chemotherapy, the patient achieved very good partial response (normal serum free light chain (SFLC) level). Repeat spine MRI and CT four months later showed resolution of the T8 lesion and new bone formation (Figure 1). Six months following her presentation, she was weaned off the brace and underwent melphalan (200 mg/m^2^) ASCT.

The majority of MM cases are diagnosed after the sixth decade of life, with only 2% being diagnosed before the age of 40.^8^ Hence, guidelines for the management of MM in pregnancy are lacking, as the simultaneous occurrence of both is scarcely reported. After the first published case of MM in pregnancy in 1965,^9^ 45 cases of MM diagnosed during or up to 3 months post-delivery have been summarised by Magen H et al.^10^ MM presenting with MSCC in pregnancy is extremely uncommon, and a similar presentation to the one described above was published in 2009.^11^ Treatment options for MM in pregnancy remain limited, due to the potential risk to the embryo and teratogenicity of the available anti-myeloma agents.^2,3^ Due to the indolent nature of MM, especially initially in the disease course, its treatment can be deferred, if there is no maternal or fetal compromise, and with the informed consent of the pregnant patient.^11^ However, MSCC is a medical emergency and, thus, urgent treatment to prevent irreversible maternal and neurological sequelae is prudent. Steroids at varying doses have been used for the treatment of MM and are effective in the management of MSCC. Non-fluorinated steroids, such as methylprednisolone and prednisolone are preferred in pregnancy, as they are 90% deactivated by placental 11 beta-hydroxysteroid dehydrogenase type 2 (11B-HSD2).^5^ The pregnancy outcome was favourable in 91% of the 45 cases described in the literature^10^ and also in the case described here. We highly recommend an MDT approach to treat these rare cases. A summary of the key proposals for managing symptomatic MM presenting with MSCC during pregnancy are outlined in Table 1.

**Table 1.** Proposed guidance for managing myeloma MSCC during pregnancy.

Close collaboration amongst MDT involving haematologist, neurosurgeon, spinal orthopaedic surgeon, obstetrician, clinical radiation-oncologist, fetal and neonatal physician to individualize patient care plan
Provide information and support about termination of pregnancy, if MM is diagnosed early in pregnancy and there are signs of end organ damage
Respect patient’s autonomy, and carefully balance maternal and foetal well-being
Close pre-natal monitoring
Assess spinal stability and need for urgent decompression surgery. MRI is safe during pregnancy, but PET/CT should be avoided
Avoid radiotherapy in late pregnancy, especially if close to the fetus. Ionizing radiation exposure of the fetus is to be limited to a cumulative dose of 100 mGy throughout the pregnancy^12^
Avoid systemic chemotherapy during first trimester when the risk of fetal harm is the greatest.
Avoid small molecule immunotherapeutic agents e.g. proteasome inhibitors (PIs), immunomodulatory drugs (iMIDs), anti-CD38 monoclonal antibodies, bispecific antibodies, CAR-T therapy.
Follow obstetric physician and surgery team’s advice regarding timing and mode of delivery.
In the postpartum period, avoid chemotherapy with high thrombogenic risk (e.g., thalidomide) especially when the patients’ mobility is poor. Ensure thromboprophylaxis with low molecular weight heparin.
Breast-feeding can be undertaken during radiotherapy but avoided while patients receive chemotherapy. However, if there is time before starting chemotherapy, pre-emptive expression and freezing of breast milk can be carried out, if the patient wishes for this.
Fit external brace following spinal orthopaedic surgeons’ and physiotherapists’ advice. Reassess the measurement of the brace to ensure the best fit. Repeat spine MRI and CT to ensure spinal stability before weaning off brace.

## Authors’ Contribution – per CRediT

KM and KX wrote up the manuscript. All the authors critically revised the final version of the manuscript.

## Conflict of Interest disclosure statement

Authors have no conflict of interest

## Consent for publication

The patient has consented for publication of this case report.

## Data sharing statement

The datasets generated during and/or analyzed during the current study are available from the corresponding author upon request.
